# Presynaptic neuromuscular transmission defect in the stiff person syndrome

**DOI:** 10.1186/s12883-016-0773-2

**Published:** 2016-12-01

**Authors:** Y. L. Lo, Y. E. Tan

**Affiliations:** 1 Department of Neurology, National Neuroscience Institute, Singapore General Hospital, Outram Road, Academia Level 4, Singapore, 169608 Singapore; 2 Duke-NUS Graduate Medical School, Singapore, Singapore; 3 Department of Neurology, Singapore General Hospital, Singapore, Singapore

**Keywords:** Stiff person syndrome, Fatigue, Presynaptic, Neuromuscular transmission

## Abstract

**Background:**

The stiff person syndrome (SPS) is a rare disorder characterized by muscular rigidity and stiffness.

**Case presentations:**

We describe an SPS patient presenting with longstanding fatigue and electrophysiological evidence of presynaptic neuromuscular transmission defect, who responded to administration of pyridostigmine. In contrast, no electrophysiolgical evidence of neuromuscular transmission defect was demonstrated in 2 other SPS patients without fatigue symptoms.

**Conclusions:**

Our findings suggest that glutamic acid decarboxylase (GAD) antibodies may play a role in presynaptic neuromuscular transmission defect of SPS patients with fatigue.

## Background

The stiff person syndrome (SPS) is a rare disorder characterized by progressive rigidity and stiffness. SPS characteristically affects the truncal or axial muscles, and may lead to chronic pain, spasms, postural deformities and impaired motility. Variants of SPS, including stiff limb syndrome, jerking man syndrome and paraneoplastic associations have been described.

Up to 80% [1] of SPS patients have elevated glutamic acid decarboxylase (GAD) antibodies, but its exact role in the pathogenesis remains unclear. GAD antibody levels generally do not correlate with disease severity [2], nor has it been determined as the sole cause of clinical manifestations.

GAD is a presynaptic molecule which exists in 2 main isoforms: GAD67 and GAD65. The latter is of interest as it is localized to the nerve terminals and synthesizes gamma-aminobutyric acid (GABA) for neurotransmission [3]. Most SPS patients with GAD antibodies also have antibodies which inhibit GABA-receptor-associated protein, leading to GABA functional impairment and hence the clinical features [4].

The occurrence of fatigue has been reported in SPS [5] but may be overshadowed by other muscular complaints. Neuromuscular fatigue is well correlated with neuromuscular transmission defect. This may be postsynaptic, as in myasthenia gravis, or presynaptic, as in the Lambert Eaton and Miller Fisher syndromes [6, 7].

We describe an SPS patient presenting with longstanding fatigue and electrophysiological evidence of presynaptic neuromuscular transmission defect.

## Case presentations

### Patient 1

A 62-year old previously healthy Caucasian female experienced a 20 year history of pain in the neck, truncal and limb muscles. The symptoms were exacerbated by exercise and had a fluctuating course. In addition, she has muscle cramps, stiffness and spasms which she needed periods of rest to allow symptoms to alleviate.

Notably, she reported severe fatigue symptoms for the same period of time, made worse with physical exertion and mental stress. She had consulted multiple doctors and given diagnoses, including chronic fatigue syndrome (CFS) or myalic encephalomyelitis, and fibromyalgia. These symptoms had severely affected her lifestyle and can last up to several days in duration.

There were no complaints of confusion, memory loss, seizures, vertigo, loss of appetite or weight.

Clinical examination showed a well and alert middle-aged woman. No signs of muscle wasting, tremor, dystonia or fasciculations were observed. There was no obvious muscle weakness or fatigability. Examination of cranial nerves, cerebellar system, sensation and position sense were unremarkable.

Nerve conduction study is not suggestive of sensorimotor polyneuropathy. Needle electromyography performed showed involuntary firing of motor units with normal morphology at rest. During voluntary activation, there was normal recruitment of motor units seen.

Stimulated single fiber electromyography (SFEMG) of the orbicularis oculi was achieved using a disposable monopolar needle electrode (TECA, Old Woking, United Kingdom) placed 2.5 cm away from the edge of the orbicularis oculi. Stimulation pulses of 0.01 ms at 10 Hz and 5 to 12 mA were administered. A 40-mm 9013K0872needle electrode (Dantec, Skovlunde, Denmark) was inserted at the edge of the muscle for single-fiber recordings. Filter settings were maintained at 500 kHz to 10 kHz. Single-fiber responses were selected on the basis of short rise times (<300 us), clear separation from other discharges and stability of waveform. Mean jitter was calculated from 20 accepted single-fiber responses. All SFEMG studies were performed on a Dantec Keypoint EMG machine [8]. SFEMG showed a mean jitter of 27.7 μs (normal < 23), of which 13 of 42 fibers recorded jitter values above 30 μs.

Repetitive nerve stimulation (RNS) studiesof the ulnar nerve was performed with right abductor digiti minimi recording [6] at the following frequencies in random order: 3 Hz at rest, 3 Hz post-exercise, 20 Hz and 50 Hz. Exercise consisted of 20 s of maximal muscle contraction. Each study was performed at 3-min intervals. The 3 Hz RNS consisted of 10 stimuli trains, while 20 Hz and 50 Hz RNS consisted of 30 stimuli trains. For 3 Hz RNS, negative peak amplitude percentage decrements were compared between the first and fourth compound muscle action potentials (CMAP). For 20 Hz and 50 Hz RNS, percentage increments between the first CMAP and the CMAP with the largest amplitude were calculated. For this patient, RNS showed amplitude decrement of −1% (normal < − 8%) at 3 Hz, increment of +71% (normal < 48%) at 20 Hz, and +67% at 50 Hz (normal < 52%).

Acetylcholine receptor and anti-MUSK antibody titres were within normal limits. Autoantibody screening showed increased antinuclear factor titre of 1/100 but anti- DsDNA, ANCA, Anti-Ro (SSA), anti-La (SSB) and anti-Jo-1 antibody titres were normal.

She was subsequently found to have elevated anti-GAD titre of 19 U/mL (normal < 0.8).

She underwent CT scan of her chest, abdomen and pelvis but no malignancy was detected. MRI of the brain and spine performed previously were unremarkable.

In view of her religious beliefs, she declined intravenous immunoglobulin (IVIg), but was administered oral prednisolone 30 mg daily over a 6 week period. This was effective in reducing muscle cramps and spasms. Pain score had declined from 6 to 2 on the visual analogue scale. To address her longstanding fatigue, a trial of pyridostigmine at 60 mg three times a day over a 4 week period resulted in significant reduction of fatigue symptoms. Using the Fatigue Severity Scale [9], initial scoring of 49 declined to 28 after treatment. On a 10 point visual analogue fatigue scale, the initial score of 8 declined to 3.5.

### Patient 2

A 63-year old man presented with back pain and stiffness radiating to the anterior trunk, neck and chest. Clinically, no involuntary movements, muscle wasting, fasciculations or tremors were noted. He was neurologically normal on examination. He was extensively investigated with brain imaging, gastroscopy, colonoscopy, CT scans of chest and abdomen which were all unremarkable. MRI of the brain was unremarkable, but there were mild degenerative changes in the lumbar spine. An autoimmune screen detected elevated anti-GAD titer of 50 U/mL. EMG showed characteristic continuous normal motor unit activity at rest in the paraspinal and shoulder girdle muscles, without myokymia or myotonic potentials.

RNS was performed using a similar method as in Patient 1. RNS amplitude decrement of −1% at 3 Hz, increment of +31.1% at 20 Hz, and +13.1% at 50 Hz, all within normal limits, were noted. SFEMG findings were within normal limits.

He responded to 2 courses of IVIg administered over 5 days but did not record benefit with oral corticosteroids. Pain score declined from 8 to 4 after each IVIg administration.

### Patient 3

A 56-year old man complained of left lower limb stiffness and cramps developing over a 2 month period, which gradually involved the right side. Upon presentation, tendon reflexes were normal but power was difficult to assess due to stiffness. Investigations to rule out malignancy were negative. Autoimmune testing showed elevated anti-GAD levels of 130 U/mL (normal up to 0.8), but other antibody titres were not elevated.

EMG gain showed continuous normal motor unit activity at rest in the paraspinal, proximal limb and shoulder girdle muscles.

CT scans of the brain, chest, abdomen and pelvis did not reveal space occupying lesions. Mild degenerative changes were present in the cervical and thoracic MRI, but brain MRI was unremarkable.

RNS was performed using a similar method as in Patient 1. RNS amplitude decrement of – 4.6% at 3 Hz, increment of +9.2% at 20 Hz, and +6.2% at 50 Hz, all within normal limits, were noted.

The stiffness responded to a 5 day course of IVIg but he experienced a relapse, requiring a second course of IVIg and oral corticosteroids. Upon review 8 weeks later, he continued to improve and oral medication dosages were reduced. Pain score declined from 8 to 3 after each treatment.

All 3 patients had unremarkable cerebrospinal fluid examination, without elevation of cells or protein.

**Fig. 1:**
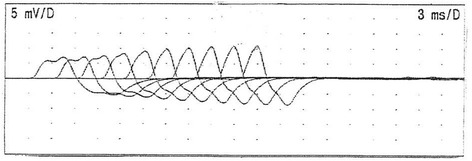
RNS tracings of Patient 1 showing maximal amplitude increment of +71% at 20 Hz. Sweep speed and vertical gain are as shown

**Fig. 2:**
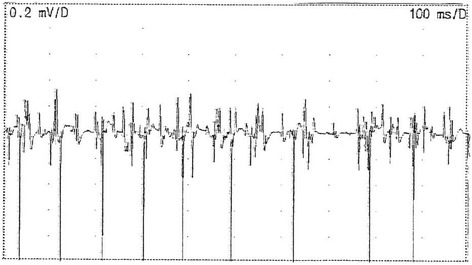
EMG of the left deltoid of Patient 1 depicting spontaneous normal motor unit activity. Sweep speed and vertical gain are as shown

Figures 1 and 2 are RNS and EMG tracings of Patient 1.

## Conclusions

Our series of 3 patients highlights the findings of presynaptic neuromuscular transmission defect in Patient 1, evidenced by symptomatic response to pyridostigmine and electrophysiological testing. In contrast, Patient 2 with classical SPS and Patient 3 with the stiff limb syndrome variant [10] both do not exhibit fatigue symptoms or positive electrophysiological evidence of neuromuscular transmission disturbance, thus serving as negative controls.

Incremental responses to RNS are characteristic of neuromuscular transmission defect at the presynaptic region [11]. We have previously reported similar findings in the Miller Fisher syndrome as a subclinical phenomenon [7]. However, the current Patient 1 is symptomatic and responded to pharmacological intervention. Fatigue is seldom reported in SPS, but Teggi et al. described an SPS patient with fatigue and recurrent vertigo who improved with immune therapy [5]. However, it is unclear if this patient had neuromuscular transmission disturbances.

GAD65 is important for GABA synthesis in normal synaptic transmission. It has been shown in a *Drosophila* model that GAD mutants can result in defective synaptic transmission at the neuromuscular junction, as GAD is specifically required in the presynaptic neuron to induce a an appropriate postsynaptic response [12].

In SPS, it is unclear the direct pathogenic role GAD antibodies play in its symptomatology. It has been suggested that reduction of GABA-mediated inhibitory effects leads to a pathological state of neuronal hyperexcitability. Injection of IgG-GAD antibodies in the lumbar region induced continuous motor activity of anterior horn cells [13].

In the *Drosophila* model [12], GAD is required in the presynaptic neuron to induce a postsynaptic glutamate receptor field, and the levels of postsynaptic receptors are closely dependent on presynaptic GAD function. Our electrophysiological findings point to a presynaptic neuromuscular transmission defect, but in human SPS, the effect of GAD antibodies is still unknown in the neuromuscular junction.

To date, GAD antibodies have been found in increased titer in Miller Fisher syndrome, a condition with a presynaptic neuromuscular transmission defect [14], and myasthenia gravis [15], where the defect occurs at the postsynaptic region. Hence, the effect of GAD antibodies may not be limited only to a single location in the nervous system.

Conversely, other autoantibodies against amphiphysin and gephyrin [16] have been reported in SPS. Their immunological effects on the central and peripheral nervous system remain to be seen in this condition.

To our knowledge, this is the first report of presynaptic neuromuscular transmission defect occurring in a patient with chronic SPS. It follows that the diagnoses of CFS, myalgic encephalomyelitis and fibromyalgia are reasonable differentials here which justify further research into the role of neuromuscular transmission derangements in their respective pathophysiology.

## Abbreviations

CFS: Chronic fatigue syndrome
CMAP: Compound muscle action potention
GABA: Gamma-aminobutyric acid
GAD: Glutamic acid decarboxylase
IvIg: Intravenous immunoglobulin
RNS: Repetitive nerve stimulation
SFEMG: Single fibre electromyography
SPS: Stiff person syndrome
